# Use of drugs in the ICU and its iatrogenic potential

**DOI:** 10.1186/cc12632

**Published:** 2013-06-19

**Authors:** É de Castro Vieira, NM Vidal, GMA Fideles, EC Santos, AA Peixoto, FA Meneses

**Affiliations:** 1 Hospital Geral de Fortaleza, Papicu, Fortaleza, CE, Brazil

## Introduction

Developed and administered for the purpose of benefit, pharmaceutical agents can cause harmful effects to the patient. We analyzed their use in a population of patients admitted to the ICU, trying to detect their potential exposure to drug-drug interactions.

## Methods

Prospective study of patients hospitalized for more than 48 hours, during the period from May to July 2012, with tracking of drug interactions according to Micromedex (Version 2.0).

## Results

We analyzed 50 patients with a mean age 45.4 ± 18.7 years, the majority were female (54%) and from the emergency unit (74%). The mean APACHE II score was 17.6 ± 7.3 points, the mean SOFA score (day 1) 7.3 ± 4.2 points, and the median length of stay 21 (IQR: 12.5 to 31.5) days. One hundred and three drugs were prescribed, predominantly antimicrobials (100% of patients) and analgesics (98% of patients). The average/day/patient of prescription drugs was 10 ± 2.6, and the average/day/patient drug interactions 2.7 ± 2.8. On exposure to drug interactions important and moderate risk occurred, respectively, in 78% and 86%, identifying the association contraindicated in 34% of patients of a positive correlation between length of stay in the ICU and exposure to important risk interactions (*r *= 0.53, *P *= 0.00006) and moderate risk interactions (*r *= 0.34, *P *= 0.013). Patients exposed to important risk interactions had greater severity at ICU admission for the APACHE II (18.5 ± 7.1 vs. 14.3 ± 6.9 points, *P *= 0.045) and SOFA (day 1) (7.8 ± 4 vs. 4.2 ± 3.9 points, *P *= 0.017).

## Conclusion

The high numbers of drug interactions with important risk incidents, especially in the sick population, alert us to the necessity of knowledge by the intensivist for the use of drugs, due to the iatrogenic potential exacerbating the severity already underway.
